# Coexistence of a Nasopalatine Duct Cyst and Radicular Cyst: A Unique Clinical Presentation

**DOI:** 10.7759/cureus.46774

**Published:** 2023-10-10

**Authors:** Muath S Alassaf, Mohannad M Abu Aof, Ahmad Othman, Hattan Zaki, Abdulaziz G Almutairi

**Affiliations:** 1 Orthodontics and Dentofacial Orthopedics, Taibah University, Madinah, SAU; 2 Dental Education, Taibah University, Madinah, SAU; 3 Dentistry, Taibah University, Madinah, SAU; 4 Oral Basic and Clinical Sciences Department, College of Dentistry, Taibah University, Madinah, SAU

**Keywords:** non-odontogenic cyst, surgical enucleation, odontogenic cysts, radicular cyst, nasopalatine duct cyst

## Abstract

This study discusses a case of coexistence of two distinct cysts, a nasopalatine duct cyst (NPDC) and a radicular cyst, within the anterior region of the maxilla. NPDC is a prevalent non-odontogenic developmental cyst, while radicular cysts are commonly found in odontogenic inflammatory cysts. The clinical and radiographic characteristics of these cysts are explored, emphasizing the importance of accurate diagnosis and treatment planning. In this case, a 51-year-old male patient presented with swelling and pain in the maxillary anterior region. Radiographic examinations revealed a heart-shaped radiolucent lesion extending from tooth 13 to 23, associated with the NPDC, and a separate radicular cyst. Surgical enucleation and tooth extraction were performed as the treatment of choice. This unique case underscores the significance of meticulous radiographic assessment to detect multiple cystic lesions within the same area.

## Introduction

The nasopalatine duct cyst (NPDC) is one of the most common non-odontogenic developmental cysts known as incisive canal cysts [1-4]. Meanwhile, the radicular cyst is one of the most common odontogenic inflammatory cysts, also called periapical cysts [5]. This particular type of non-odontogenic cyst originates from the embryogenic remains of the nasopalatine duct [1-3,6]. However, the radicular cyst is due to the proliferation of epithelium caused by inflammation from necrosis of the tooth pulp [5]. NPDC is located at the midline of the anterior palate behind the maxillary incisors [1-3]. A radicular cyst is related to the apex of the non-vital tooth [7].

Furthermore, NPDC can be developed at any age but is more common in the fourth to sixth decades of life, and in the case of radicular cysts, it is more frequent between the second and fifth decades of life with more incidence in the male gender for both lesions [2,4,7-9].

According to some literature works, the cyst clinically appears as swelling, usually asymptomatic in the anterior region of the palate, which is detected by routine clinical check-ups or radiographs [1-3,6,10]. Nevertheless, additional symptoms may be linked to this particular lesion, such as the presence of drainage and discomfort resulting from the compression of neighboring tissues, such as the nasopalatine nerves. These symptoms may arise in cases of over-infection or prior infection of the lesion [3,6,10]. In addition to these symptoms, displacement of the nearby teeth or burning sensation in the affected region with radiated pain could be the complaint in rare cases [2,10].

In the case of radicular cysts, although they could be asymptomatic and discovered by periapical radiograph while diagnosing a non-vital tooth, the peri-radicular inflammation can lead to many symptoms, such as swelling, pain, abscess, or intraoral or extraoral fistula [7].

Radiographically, NPDCs show a well-defined rounded or heart-shaped radiolucent area surrounded by a sclerotic margin. Moreover, the intact status of lamina dura around the nearby teeth apices was confirmed; however, according to Allard et al., resorption of maxillary incisors root might be seen in rare cases [1-3]. Even though the radicular cyst appears to have a defined rounded or oval radiolucency, it is related to non-vital tooth apex with loss of the continuity of lamina dura, unlike NPDCs [7].

Through the histopathological examination, NPDCs appear as a cavity with an epithelial lining surrounded by a connective tissue wall. Moreover, the presence of mucous glands and neurovascular bundles, in addition to variable types of the epithelium, can be seen, such as columnar, cuboidal, squamous, or even respiratory type of epithelium, depending on the proximity to the nasal cavity [2,3,8]. By contrast, the radicular cyst histologically shows a cavity lined by non-keratinized stratified squamous epithelium surrounded by a connective tissue wall with variable contents, such as inflammatory cells infiltrate and cholesterol clefts [7,11].

The standard treatment modality of NPDCs is surgical by enucleation, which is indicated for cases with clinical symptoms that interfere with their quality of life or for diagnostic purposes in the case of extensive lesions [2,3]. Meanwhile, inflammatory lesions of the periapical area are treated by endodontic therapy; however, the failure to relieve signs and symptoms makes surgical enucleation a treatment of choice for radicular cysts [7,11].

This case report presents a case of the coexistence of two distinct cysts with different origins and pathogenesis in the anterior maxilla.

## Case presentation

History and examination

**Figure 1:**
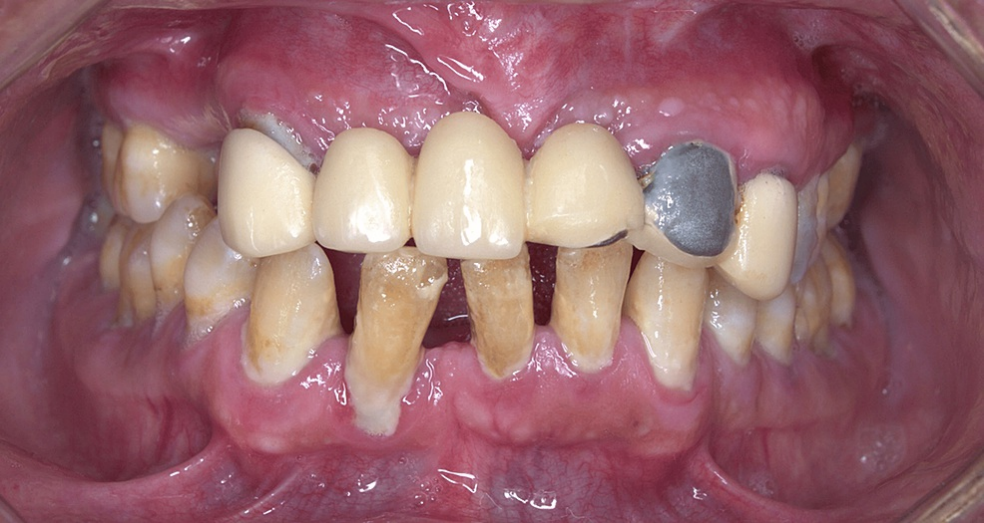
Pre-operative frontal picture showing the upper anterior bridge

A 51-year-old male patient came to the dental clinic with a chief complaint, “I want to repair my anterior bridge,” with no significant findings regarding medical and social history; however, the patient had a history of trauma on the upper anterior area due to a car accident 10 years ago, which led to multiple extractions and fabrication of the upper anterior bridge from the upper right canine to the upper left canine. Furthermore, the patient complained of pain and swelling that started several weeks ago in the maxillary anterior teeth region (Figure 1).

**Figure 2:**
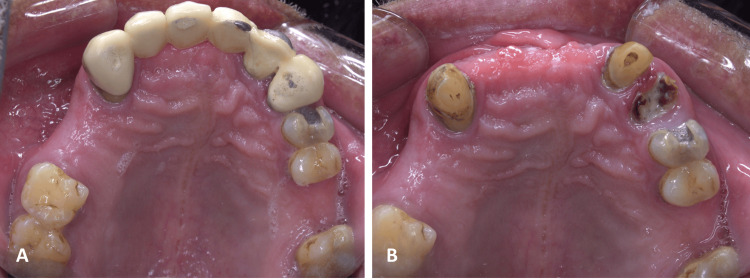
Operative occlusal photograph showing palatal swelling in the right area of the hard palate before bridge removal (A) and after removal of the bridge (B)

Upon intraoral clinical examination, there was tender, firm swelling on the right side of the anterior area of the hard palate that crossed the midline to the area of the left maxillary central incisor with normal covering mucosa (Figure 2A, 2B). Moreover, the patient has a missing maxillary right and left central incisor in addition to the maxillary right lateral incisor, all replaced by a fixed long-span bridge. Fine-needle aspiration with an 18-gauge syringe of the swelling content revealed red-brown thin fluid, in addition to multiple missing posterior teeth.

Radiographic examination

**Figure 3:**
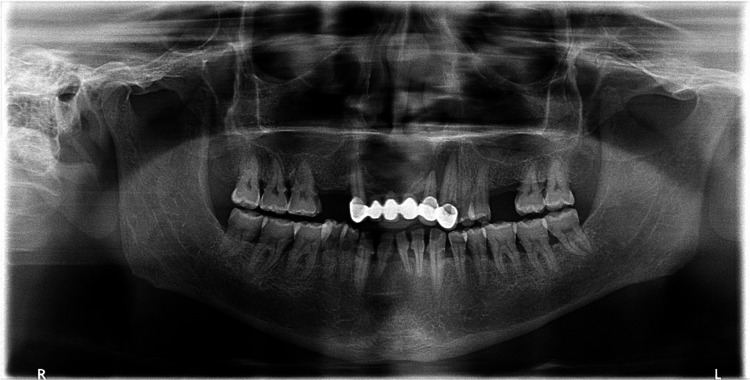
Orthopantomogram showing a heart-shaped lesion in the anterior maxilla

**Figure 4:**
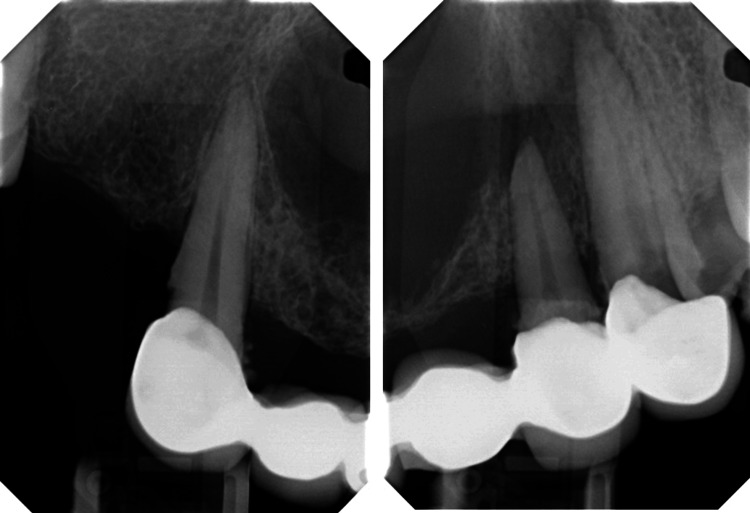
Periapical radiograph of the teeth associated with the cysts

**Figure 5:**
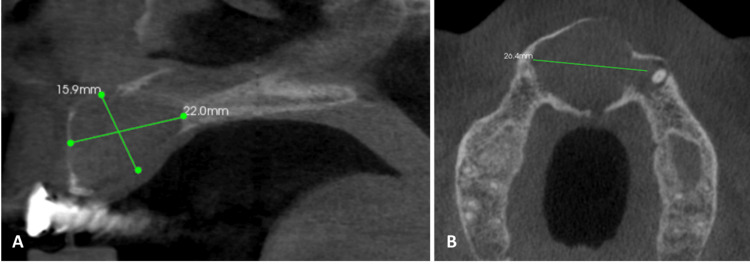
Cone-beam computed tomography of the case showing the nasopalatine duct cyst (NPDC) in sagittal (A) and axial (B) cuts

On radiographic examination, orthopantomography (OPG) shows a well-defined heart-shaped radiolucent lesion extending from tooth #13, crossing the midline to tooth #23 with loss of the continuity of lamina dura of the teeth related to the lesion (Figure 3). The periapical radiographs showed similar findings as in OPG (Figure 4). Three-dimensional radiographic examination with cone-beam computed tomography revealed a well-defined hypodense lesion measuring 22 mm anterior-posterior and superior-inferior 15.9 mm and medio-lateral 26.4 mm. The lesion perforated the bone to the nasal floor and labially to the mucosa (Figure 5).

Incisional biopsy

Further investigation was needed, so an incisional biopsy under local anesthesia from the labial vestibule in the apical area of the upper left canine was done, and the specimen was sent for histopathological examination. It shows dense fibrous connective tissue with chronic inflammatory infiltrates containing predominantly lymphocytes and plasma cells. Moreover, there are few areas of hemorrhage and small blood vessels. The diagnosis was a periapical cyst based on the histopathological findings.

Management

The preferred therapeutic intervention involved surgical enucleation of the cyst, coupled with the extraction of the nonviable upper left lateral incisor and canine. Under local anesthesia, by performing bilateral greater palatine nerve blocks and bilateral anterior superior alveolar nerve blocks, the previously placed defective anterior bridge was removed. Subsequent to this, the patient was directed to rinse with a chlorhexidine mouthwash. Both crestal and sulcular incisions were executed, accompanied by two vertical releasing incisions. A periosteal elevator was utilized to reflect the flap. The cystic wall was then carefully dissected from both the labial mucosa and the nasal floor with sharp scissors. The cyst was enucleated and preserved in formalin for histopathological evaluation. The compromised teeth were then extracted. Hemostasis was achieved, followed by the nose-blowing test to confirm the integrity of the nasal floor. The surgical site was sutured using a 4-0 polyglactin absorbable suture.

**Figure 6:**
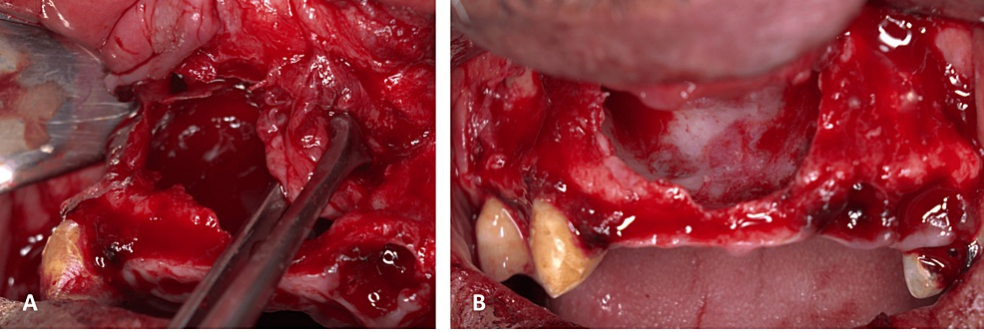
Intra-operative photograph showing the exposure of the cysts during dissection (A) and after complete enucleation of the cyst (B).

**Figure 7:**
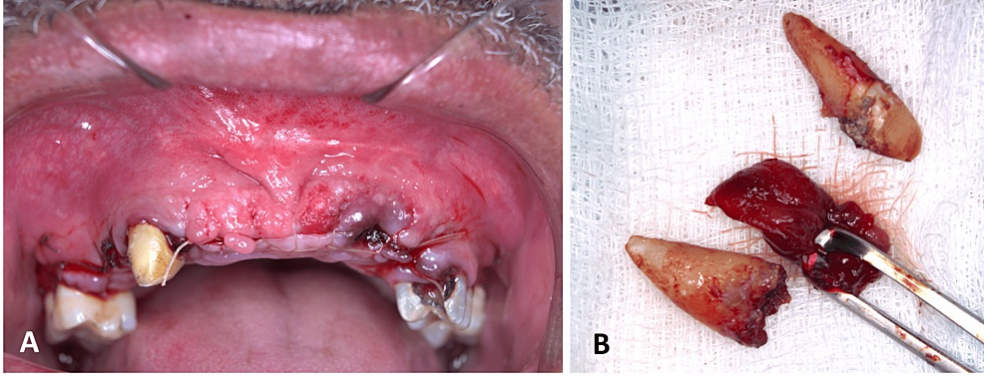
Clinical photograph after closure (A) and the extracted teeth with the gross specimen (B)

**Figure 8:**
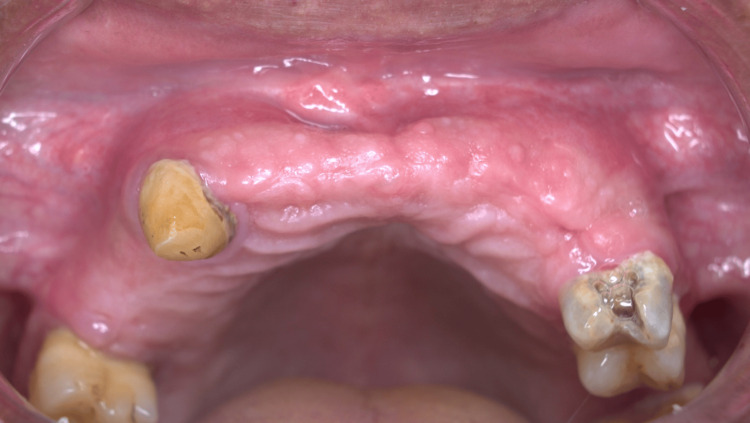
Clinical photograph of the case after two months' follow-up

**Figure 9:**
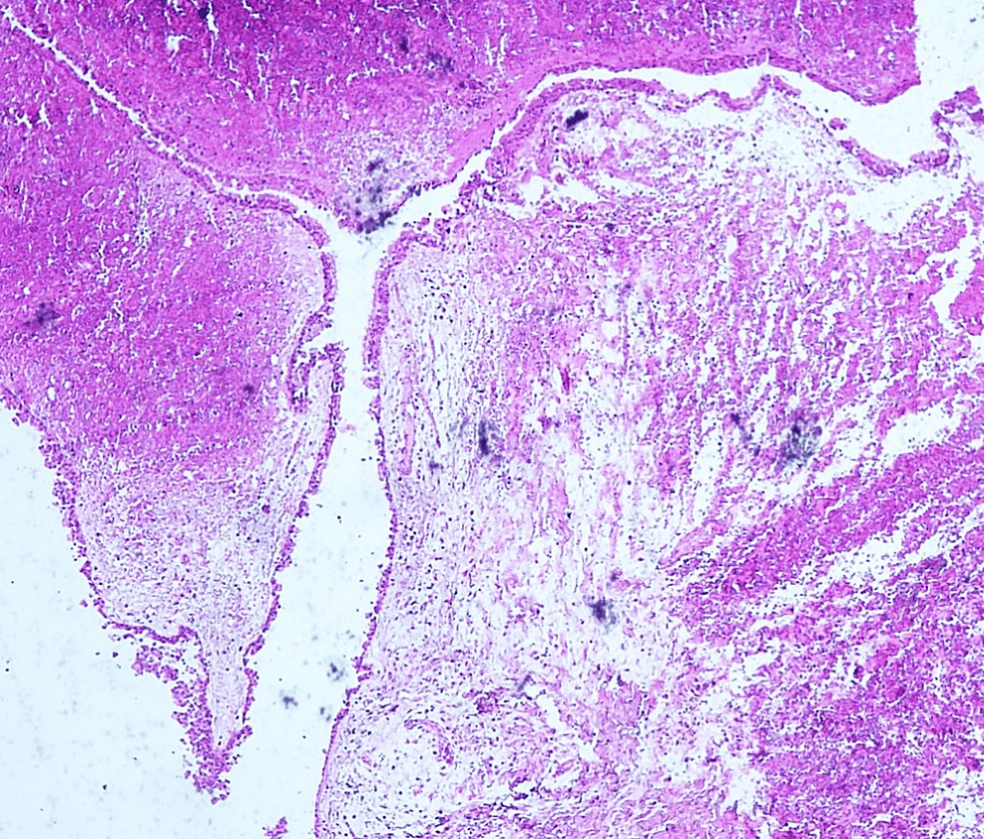
Photomicrograph of the nasopalatine duct cyst showing a pseudostratified squamous epithelium

Histopathological assessment of the removed lesion unveiled a cystic structure. This was lined by ciliated pseudostratified columnar epithelium. The underlying connective tissue was characterized by fibroblasts, collagen fibers, vasculature, and nerve fibers. Some regions also exhibited hemorrhage. Based on these findings, the final diagnosis was an NPDC. The aforementioned surgical procedures and the histopathological specimen are represented in Figures 6-9.

A meticulous re-evaluation of the periapical radiographs and CBCT revealed the presence of not one, but two cystic structures, as illustrated in Figure 4 and Figure 5B. A post-operative follow-up after two months exhibited complete healing in the treated region, as shown in Figure 8.

## Discussion

The oral cavity, with its complex embryonic development, frequently presents various pathologies. An NPDC emerges as the prevailing non-odontogenic developmental cyst, originating from the remnants of the nasopalatine duct. Radiographically, NPDCs often display a distinctive heart-shaped radiolucency situated between the central incisors [3]. By contrast, the radicular cyst, primarily inflammatory in nature, arises from the epithelial cell rests of Malassez due to the necrotic dental pulp, commonly from untreated dental caries or traumatized tooth [11]. In a study including 344 NPDCs, intraoral swelling was reported in 52% of the cases, same as in the presented case [12]. On histopathologic examination, the NPDC showed respiratory epithelium as in most cases which is also in line with this case. The clinical, radiographic, and histopathologic features of the presented case are consistent with the literature findings. While both cysts individually present a familiar scenario in oral pathology, this particular case highlighted their concomitant occurrence in the same region without any intervening bony septum - an unprecedented combination not documented in previous literature. The sheer rarity lies in the juxtaposition of these two common cysts, making this case an intriguing addition to oral pathology diagnostics.

In managing the unique concomitant occurrence of the NPDC and radicular cyst, an integrated approach is imperative. Initial diagnostic steps include comprehensive radiographic examinations, where panoramic views offer a preliminary understanding while advanced modalities, such as CBCT, provide detailed insights into the cysts' size, boundaries, and relationship to adjacent vital structures [13]. Post-surgical enucleation, it is crucial to undertake a histopathological examination of the cystic tissue. This not only confirms the diagnosis but also rules out any malignancies or other pathologies [14,15]. Given the adjacency of these cysts, meticulous surgical planning ensures complete removal, reducing the risk of recurrence. Post-operatively, patients should be closely monitored with periodic radiographs to detect any signs of recurrence or complications. The unique juxtaposition of these cysts underscores the need for a thorough diagnostic and management protocol, emphasizing the importance of both surgical precision and post-operative care.

## Conclusions

The simultaneous presence of an NPDC and a radicular cyst, in this case, serves as a testament to the unpredictable nature of oral pathologies. This unprecedented co-occurrence not only challenges the conventional understanding of cystic development but also underscores the importance of comprehensive diagnostic measures and meticulous treatment planning. The rarity of such a contiguity reinforces the importance of continual learning and adaptability in oral pathology diagnostics. As the field continues to evolve, such unique cases emphasize the importance of a holistic approach to patient care, ensuring that both surgical interventions and post-operative monitoring are conducted with utmost precision and diligence.
